# Considerations For Optimizing Microbiome Analysis Using a Marker Gene

**DOI:** 10.3389/fnut.2016.00026

**Published:** 2016-08-08

**Authors:** Jacobo de la Cuesta-Zuluaga, Juan S. Escobar

**Affiliations:** ^1^Vidarium – Nutrition, Health and Wellness Research Center, Grupo Empresarial Nutresa, Medellín, Colombia

**Keywords:** gut microbiome, 16S rRNA, next-generation sequencing, personalized medicine, personalized nutrition

## Abstract

Next-generation sequencing technologies have found a widespread use in the study of host–microbe interactions due to the increase in their throughput and their ever-decreasing costs. The analysis of human-associated microbial communities using a marker gene, particularly the 16S rRNA, has been greatly benefited from these technologies – the human gut microbiome research being a remarkable example of such analysis that has greatly expanded our understanding of microbe-mediated human health and disease, metabolism, and food absorption. 16S studies go through a series of *in vitro* and *in silico* steps that can greatly influence their outcomes. However, the lack of a standardized workflow has led to uncertainties regarding the transparency and reproducibility of gut microbiome studies. We, here, discuss the most common challenges in the archetypical 16S rRNA workflow, including the extraction of total DNA, its use as template in PCR with primers that amplify specific hypervariable regions of the gene, amplicon sequencing, the denoising and removal of low-quality reads, the detection and removal of chimeric sequences, the clustering of high-quality sequences into operational taxonomic units, and their taxonomic classification. We recommend the essential technical information that should be conveyed in publications for reproducibility of results and encourage non-experts to include procedures and available tools that mitigate most of the problems encountered in microbiome analysis.

## Introduction

The gut microbiome, our “second genome,” is the most intimate connection we have with the environment. During the last decade, the study of the gut microbiome has revolutionized our understanding of human health and disease, metabolism, and food absorption. This research field has gone beyond being a mere object of study and is now recognized as an object of intervention (1) that may eventually assist in personalized diagnostic assessment, risk stratification, disease prevention, treatment decision-making, and patients’ follow-up (2).

The gut microbiome is the target of therapies for gastrointestinal diseases, such as infection by *Clostridium difficile* or inflammatory bowel disease, metabolic conditions, such as obesity and diabetes, and non-gastrointestinal pathologies, like allergy and autism (3–5). Dietary manipulation through supplementation with pre- and probiotics, and the modulation of the microbial community with antibiotics or fecal matter transplants have been studied (6, 7) and successfully applied (8). *In vitro* models that simulate the gastrointestinal tract and that allow the fine tuning of physicochemical conditions have been developed to test the effect of different substances on particular bacterial species or even the whole microbial community (9, 10).

However, understanding how the gut microbiome contributes to the pathogenesis of complex disorders or to nutrient absorption will critically depend upon the accuracy with which we characterize this microbial community. Next-generation sequencing (NGS) technologies (11–13) are currently of wide use to this end because of their capacity to measure non-cultivable organisms, relatively low cost, and high throughput. NGS platforms have allowed measuring microbial diversity with an ever-increasing throughput and read length (14, 15) and at a constantly decreasing cost (16), which has granted the possibility for a new wave of researchers to get involved in projects of considerable size and complexity, to carry sophisticated quantitative evaluations and to study low-abundance microorganisms. The outstanding increase in the number of publications in recent years (2,319 papers published in 2015; source: Scopus) is a proof of this. It raises, nonetheless, questions about how aware all these researchers are about pitfalls in microbiome analyses.

**Figure 1:**
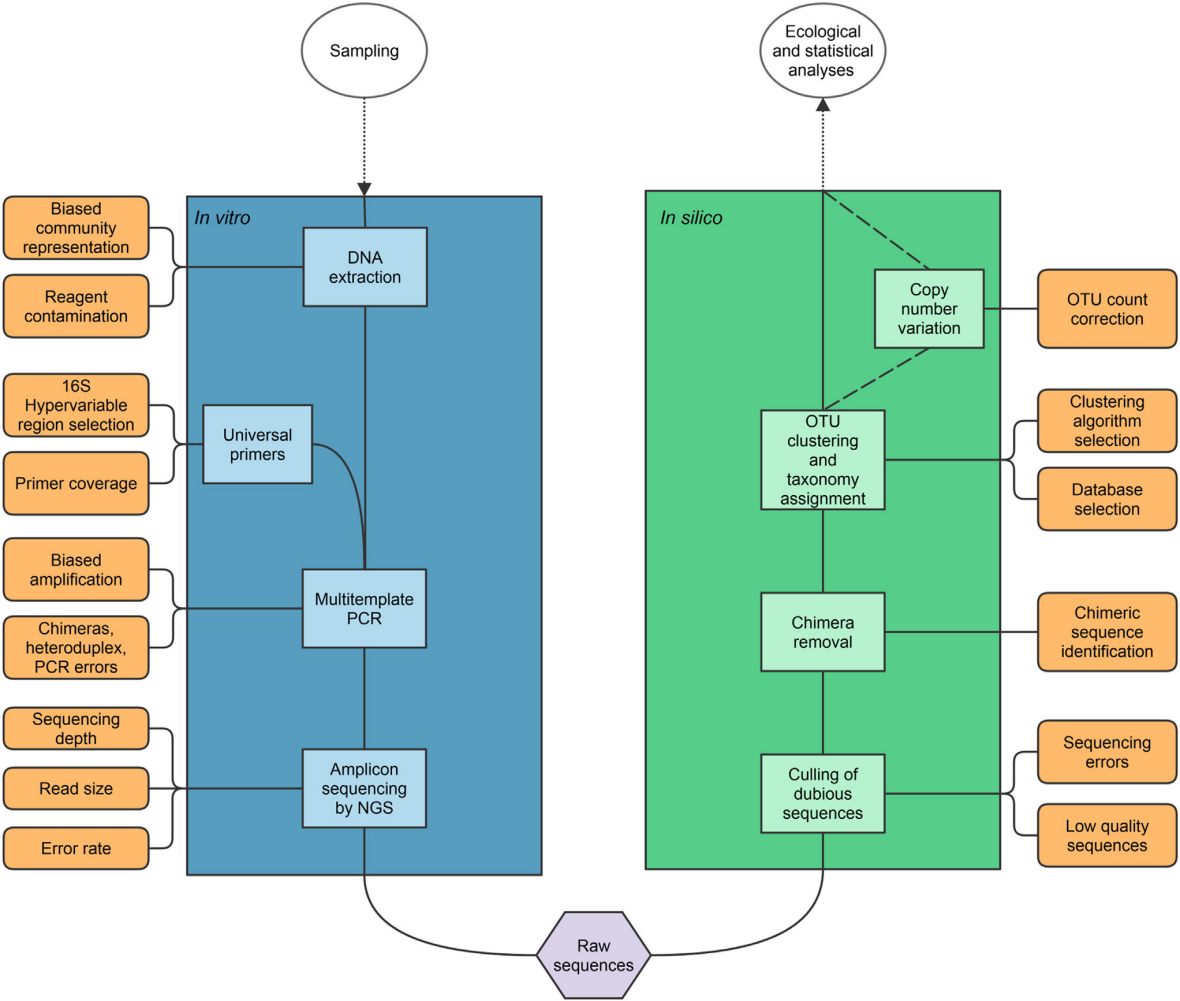
**Schematic view of the archetypical workflow in 16S rRNA studies, and some of the problems associated with each step**. Dotted lines link the workflow with steps beyond the scope of the review, and dashed lines represent non-standard steps.

One of the most used ways to examine the gut microbiome is to use a marker gene or barcode to identify microorganisms and reconstruct their phylogenetic relationships; the 16S rRNA gene is the most used for that purpose, although others have been proposed and used (17–19). As shown in Figure 1, most 16S studies follow a common workflow (20): total DNA is extracted from a sample (e.g., feces in the case of the gut microbiome) and used as template in PCR with primers that amplify specific regions of the 16S rRNA gene; the PCR products are sequenced using any technology (formerly Sanger but more recently NGS platforms, such as Roche 454, Illumina, Ion Torrent, PacBio) and raw sequences are processed using bioinformatic pipelines that include the denoising and removal of low-quality reads, the detection and removal of chimeric sequences, the clustering of the curated sequences into operational taxonomic units (OTUs), and their taxonomic classification. The output data can then be used to perform ecological and statistical tests (e.g., α and β diversity analyses). A careless execution of any single procedure in the workflow and the cumulative effect of the inherent bias of each step, which can be reduced but not totally eradicated as we shall see, can result in a biased representation of the microbial community under study or erroneous estimations of the changes induced by interventions.

The unification of analysis procedures and the implementation of standardized workflows in order to minimize the variation introduced to the results have been recurrent topics on symposia (21), editorials (22), and opinion papers (23, 24). We, here, go over each step in the workflow of an archetypical 16S study, from DNA extraction to the generation and classification of OTUs, briefly explain their principles, draw attention to their potential biases and propose some solutions to (reasonably) mitigate them, including available software tools. In addition, we highlight instances where direct comparisons between studies are discouraged and recommend the essential information that should be included when describing a microbiome study for reproducibility of results.

While some of the issues discussed here have been separately reviewed elsewhere [benefits and problems of barcode sequencing (36), primer selection (37), DNA extraction and PCR biases (38), sequence curation (39), taxonomic classification (40)], they have frequently been overlooked in publications of original datasets. We wish to encourage newcomer scientists to implement rigorous analyses so that they get confident results that better represent the microbial communities under scrutiny. Upstream and downstream procedures, namely, experimental design and sample collection, calculation of diversity indices, rarefaction curves, hypothesis testing, and other ecological and statistical analyses are of the uttermost importance; however, they vary between different kinds of studies and are beyond the scope of this paper. They have been reviewed elsewhere (41–45).

### DNA Extraction

The first step, once the samples are collected, is the extraction of total DNA, which will then be used as template for PCR amplification of the marker gene. After the DNA is extracted and purified, the workflow for most 16S studies becomes roughly the same. Fecal samples are composed of microorganisms that differ in characteristics, such as size and cell wall composition, and that are present in different proportions. This can make the purification of a DNA sample that accurately represents the original community (i.e., that keeps all species and their abundances at the same relative proportions) a challenge, as different sample handling and DNA extraction protocols can yield samples with different bacterial ratios. It has been shown, for instance, that frozen fecal samples yield a higher amount of DNA from Gram-positive than from Gram-negative bacteria, probably due to the effect that the freeze–thaw cycle can have over the Gram-positive cell wall (46).

Differences in gut microbial community patterns can also arise due to the principles of the genetic material extraction protocols, causing the over or underrepresentation of the same microbial group in DNA extracted from subsamples of the same source (47). Some DNA extraction kits use bead-containing lysing matrices and vigorous shaking steps that contribute to the disruption of the cell wall, whereas others rely on chemical lysis (48). Several studies have consistently demonstrated that protocols that involve a bead-beating step yield higher quantities of bacterial DNA, and, most importantly, these samples tend to be a more comprehensive representation of the microbial community, regardless of the source material and analysis method (49–51). The differences between subsamples extracted with different kits can even be statistically significant, which is why it has been suggested that data from studies using different extraction methods should not be compared (52). Opportunely, studies are increasingly using similar DNA extraction protocols. For instance, the PowerSoil^®^ DNA isolation kit (MoBio) has become popular because it performs well in a wide variety of samples, including human feces. Although using the same extraction protocol does not guarantee accurate representation of the microbial community under study, it allows comparison among studies.

Another issue with DNA extraction is that, due to the non-specificity of marker gene and metagenomic assays, they are highly sensitive to contamination with foreign microbial DNA. The presence of bacterial DNA from sources other than the original sample can alter the outcome of the analysis in a way that it no longer mirrors the original community it is supposed to reflect. Contamination sources may include the PCR reagents (53, 54), ultra pure water (55, 56), and, even, the DNA extraction reagents (57, 58). The genetic material extracted from samples with low biomass is more prone to being drowned by contaminant DNA (59, 60), and the contamination profile varies between laboratories, extraction kits, and batches from the same kit (60). Procedures to reduce the effect of contamination include the maximization of starting biomass from which DNA is extracted, the randomization of the order in which samples are to be processed, the collection, processing and sequencing of technical controls of the reagents to be used (storage media, DNA extraction kits, and PCR kits), the recording of the kit lots as additional metadata, and the quantification of negative-control sequences (60).

Today, there is no standard procedure on how to deal with sequences showing up in technical controls. One suggestion would be to compare the abundance in real samples and controls: if an OTU has similar relative abundance in samples and controls, it is likely a contaminant; otherwise, it probably is not. This approach has the drawback that the threshold in which the abundance of an OTU is considered a contaminant is subjective (61). Another method involves the removal of OTUs whose abundance is negatively correlated with amplicon concentration, as it is assumed that the signal from contaminant sequences in low biomass samples is less likely to be drowned by the signal of real data (61). In any case, it is necessary to be aware of taxa that are present in negative controls, taxa statistically associated with a particular batch of reagents, and taxa biologically unexpected in the treated samples.

### Multi-Template PCR

In marker gene studies, total DNA is used as template for the PCR amplification of the barcode region. As in single-template PCR, the efficiency of multi-template PCR is influenced by the GC content of the target region (62), the DNA concentration (63), and the thermocycling conditions (64). However, because of the multiorganismal origin of the gut microbiome, a series of particular difficulties and artifacts, such as primer mismatches, gene copy number variation (CNV), chimeras, heteroduplex, and skewed template-to-product ratios, are encountered and can distort the diversity measures. Primer selection, CNV normalization, and chimeric sequence removal are discussed below; for a detailed discussion of reagents and PCR conditions in multi-template assays, see Ref. (65).

#### 16S rRNA Gene Hypervariable Regions

Due to its ubiquity in prokaryotes, low horizontal gene transfer, and ability to differentiate closely related organisms, the 16S rRNA gene has been used for decades in the study of diversity and ecology of microorganisms (66–68). However, most NGS platforms are not capable of covering the full length of the gene (ca. 1,500 bp) (68). This is why short regions within the gene (e.g., hypervariable V1–V9 regions) have been prioritized with the advent of these newer technologies (69). Hypervariable regions are supposed to act as proxies of the complete gene. Actually, there is correlation between the phylogenies generated using different hypervariable regions or combinations thereof and the phylogenies generated with the whole gene (69), but the strength of these correlations varies among regions (70) because their different evolutionary rates limit their capacity to serve as surrogates of full-length sequences (71, 72). Because of these disparities, the OTU count of different 16S regions can be inconsistent (70, 73), which, in turn, makes studies using different hypervariable regions incomparable (71). Currently, there is no consensus of which region best reflects the gut microbial community (69, 74, 75). While read length increases in newer NGS technologies, one empirical way to overcome comparability between studies would be to sequence the same hypervariable region. This is, indeed, what is seen in many gut microbiome studies today: since the Illumina MiSeq platform gives one of the bests value for money of all NGS, most microbiome researchers are moving to sequence the V4 region since its size (ca. 250 bp) fits well the read size of this platform at its current version.

#### Primer Selection

In order to amplify the selected 16S hypervariable region, a set of broad-range primers (so-called “universal primers”) must be used. These primers are usually designed to hybridize with the conserved regions flanking the sequence of interest. Universal primers work under the assumption that the flanking regions are conserved among a wide range of microbial groups, which allows the correct annealing and amplification of the desired PCR product (76). The rationale behind this approach is as good as possible but it still has problems, as mutations also occur within the flanking regions. The use of primers with a suboptimal coverage rate can lead to selective amplification of the template DNA, that is, the sub-representation or selection against a given microbial group (77). Thus, the relative content of sequences may be modified, resulting in a deviation from the true gut-community composition (77–79).

In short, studies evaluating biases introduced by primer selection have demonstrated that there is no such thing as a truly “universal primer,” since there is no single pair of primers that can be used to amplify all prokaryotic or even bacterial groups. Genome evolution being what it is, the practical way to overcome this limitation and compare results among studies is to use similar pairs of primers and allow for degenerate sites in them. This is the preferred approach in some recent studies that make extensive use of modified 515F (5′ GTGYCAGCMGCCGCGGTAA 3′) and 806R (5′ GGACTACNVGGGTWTCTAAT 3′) primers that amplify the V4 region (80–82).

### Amplicon Sequencing by NGS

Next-generation sequencing technologies refer to various strategies that rely on a combination of template preparation, sequencing and imaging, and genome alignment and assembly methods (83). The major advance offered by NGS is the ability to produce an enormous volume of data cheaply and fast. The transition from Sanger to NGS has opened new horizons in the gut-microbiome field by making it possible to collect millions of sequences, spanning hundreds of samples (80). A good example of this is the Human Microbiome Project, which used NGS to characterize the diversity of bacteria, archaea, and viruses that inhabit various areas of the human body in several hundreds healthy individuals (84). In the last decade, the throughput of NGS technologies has dramatically increased, and the operation cost has reduced, which, in turn, has boosted its use in microbial studies. However, the major drawback of all NGS technologies is that they raise concerns regarding the quality of data.

When sequencing genomes, multiple reads are used to construct a consensus and the error rate, defined as the number of errors per total base call (25), is, thus, reduced since each nucleotide in the original sequence is called several times by different reads. Such approach cannot be used when sequencing marker gene amplicons, such as the 16S rRNA, because each individual read is considered an identifier of an independent organism (e.g., a bacterium), and it is not possible to assemble the amplicon sequences (34); hence, the reduction of the error rate by other means becomes imperative.

One strategy to determine how many errors are introduced at each NGS run consists of sequencing a synthetic mixture of genomic DNA (mock community), comprising several known bacterial species, along with the samples. Reads are compared with a reference database of the marker gene, and errors are identified in pairwise alignments of each experimentally generated sequence relative to the closest reference sequence (25, 32, 34). Sequencing mock communities to assess the error rate of each individual amplicon sequencing run should become a standard step in microbial community analysis (see http://www.hmpdacc.org/HMMC/) (25, 29).

Currently, Roche 454 GS-FLX, Illumina MiSeq, Ion Torrent PGM, and PacBio SMRT are the most used platforms for the study of the gut microbiome (35, 85–87). However, each technology performs differently in the trade-off between read length, sequence throughput, and error rate (Table 1). As mentioned above, since hypervariable regions correlate differently with the whole 16S rRNA gene (88, 89), it is arguably better to sequence shorter reads at greater depths and with lower error rates (e.g., Illumina, Ion Torrent) than longer reads with higher error rates (e.g., PacBio) (34). The former allows the detection of low-abundance microorganisms (90, 91) and the avoidance of unnecessary greater computing times due to the description of non-existent organisms caused by artifactual sequences. Although increased read length usually improves classification, platforms such as PacBio are currently limited by their high sequencing error and low yield of sequencing data relative to the other platforms (34).

**Table 1:** **Specifications of the most commonly used sequencing platforms in microbial community characterization studies**.

Platform	Raw ER^a^ (%)	ER after denoise^a^ (%)	Read length (bp)	Throughput (Gb/run)	Cost/Gb (USD)	Known problems	Reference
454 FLX Titanium	1.0–2.0	<0.02	450	0.4	15,500	High error rate in homopolymer regions. Sequence quality decreases in a lengthwise fashion. Soon to be phased out	(16, 25–28)
Illumina MiSeq v2	0.8–1.0	<0.02	2 × 250	7.5	142	Sequence quality decreases in a lengthwise fashion. The second read has a higher error rate than the first read. Increased single-base errors in association with GGC motifs	(16, 26, 29–31)
Ion Torrent PGM 316 chip	1.5	NA^b^	400	1	674	Premature sequence truncation caused by organism- and orientation-dependent biases. Low accuracy in homopolymer regions	(16, 31–33)
PacBio RS II	1.8	0.3	10,000	0.1	1,100	Systematic and non-random errors; G and C are more likely to be deleted than A and C. Preferential loading of shorter sequences into zero-mode waveguides	(16, 27, 31, 34, 35)

*^a^Error rate calculated by sequencing of 16S amplicons from mock bacterial communities*.

*^b^To the best of our knowledge, there are no available studies assessing the error rate of Ion Torrent sequences after bioinformatic curation*.

### Culling of Dubious Sequences

Up to this point, procedures in the archetypical workflow described in Figure 1 take place *in vitro*. Hereafter, treatment of raw DNA sequences occurs *in silico*. To reduce sequencing error rates, it has become mandatory to apply stringent sequence curation and denoising algorithms. Inadequate cleaning of reads can have many negative effects including limited ability to identify chimeras and inflation of α and β diversity metrics (92). Low-quality sequences, artifacts, and contamination can compromise the downstream analyses and, thus, must be removed from the dataset.

The first step is the removal of reads with ambiguous base calls (N) in the barcode or in the marker gene amplicon, as it is not possible to determine the true nucleotide sequence (93). On the other hand, mismatches in the primers and barcodes are usually allowed up to a certain number; the removal of sequences with less than three mismatches has little effect on the reduction of the error rate (93). Emulsion-PCR-based platforms (e.g., 454, Ion Torrent) are known for producing homopolymer-associated indel errors (33); these artifacts have been shown to account for a large proportion of errors in benchmark studies using mock communities and to be associated with low-quality scores (92). Therefore, reads with homopolymers longer than eight nucleotides should be culled (25).

In addition, in most sequencing platforms (e.g., 454, Illumina, Ion Torrent), quality scores reduce in a lengthwise fashion, and it is possible to identify breakpoints where the quality criteria are not met. Sequences can be trimmed to those breakpoints to reduce the overall error rate. Two trimming approaches have been widely used: a “hard cutoff” method trims the sequences at the first nucleotide with a quality score below a given threshold (94); this minimizes the error rate but also reduces the average sequence length. Another method, called “sliding window,” calculates the average quality score within a sequence window (or substring) and trims when the average quality score within that window drops below a threshold; the latter method has the advantage that reduces the overall error rate without reducing the average sequence length (25). Reads with anomalous lengths (well above or below the expected value for a given technology) are also removed, as they likely represent PCR or sequencing errors, or become not informative as a result of the quality trimming (93).

The use of a pre-clustering algorithm has also been shown to reduce the number of sequences that are the result of sequencing errors and to predict with higher accuracy the number of expected OTUs in template preparations of known taxonomic composition (95). It assumes that rare sequences are more likely to derive from abundant sequences and can, therefore, be merged if they are within a specified similarity threshold. This threshold must always be lower than the value used for OTU clustering, usually 1% (25).

Also, contaminant sequences must be removed from the dataset. Due to the nature of the 16S rRNA gene, mitochondria, chloroplast (96), and other eukaryotic sequences are likely to be amplified and should be identified and discarded, along with sequences unclassified at the domain level; according to the scope of the study and the primers used, bacterial or archaeal sequences would also be needed to get removed.

### Chimera Removal

Sequences composed of two or more parents are named chimeras. Chimeras are a serious concern in studies of the gut microbiome because they can lead to the description of non-existent organisms and inflate diversity metrics. This kind of artifact arises from errors during PCR, and several factors influence its appearance, such as DNA damage (97), the amplification of highly similar sequences (98), a high number of cycles, and short elongation times (99). This suggests that prematurely terminated amplicons that anneal to a homologous template to prime the next PCR cycle are likely to be the major cause of chimera formation.

The detection of chimeras in libraries of 16S amplicons is particularly challenging, as sequences are short and highly similar. There are multiple algorithms designed to detect and remove chimeric sequences (100–107), which follow the same basic principle: substrings or fragments of the query sequence are compared to a set of reference sequences in order to establish if the said substrings match different references. Once a chimera is identified, it is removed from the dataset. Some algorithms use allegedly chimera-free 16S sequence databases as reference, including Chimera Slayer (105) and DECIPHER (108). Others [e.g., Perseus (106), UCHIME (107)] use a database-free approach that assumes that the most abundant sequences from the query dataset are unlikely to be chimeric and can, therefore, be used as reference. Database dependency influences the ability of different algorithms to identify and remove chimeras (109). Database-independent algorithms have the advantage of being able to detect them even if the studied community is poorly described (25). In contrast, database-dependent algorithms rely on reference collections that only contain gene sequences from cultured bacteria and are not expected to perform as well on samples that contain sequences from yet uncultured organisms (24), something very common in studies of the gut microbiome. Thus, the use of algorithms that do not rely on databases should be preferred in order to minimize the inflation of diversity caused by chimeras, especially when dealing with poorly characterized gut microbial communities.

### OTU Clustering and Taxonomy Assignment

#### Sequence Grouping

In order to describe and compare gut microbiomes or shifts in the gut microbiome following intervention, diversity metrics should be estimated (e.g., Chao-1, UniFrac), which requires information about the composition and abundance of organisms in said communities. Currently, two approaches are used to characterize microbial communities: taxonomic-dependent (also called phylotype analysis) and OTU-based methods (110).

The taxonomic-dependent methods rely on reference databases of full-length 16S rRNA gene sequences from cultured microorganisms (i.e., with a known taxonomy). Some popular reference databases are Greengenes (111), SILVA (112), and RDP (113). Query sequences are compared against the reference database and assigned to the organism of the best-matched reference (114). While this approach is computationally fast and allows the straightforward taxonomic labeling of a query sequence, indicating its relationship to previously characterized microorganisms, it is hindered by the lack of well-annotated or incomplete databases (115). This is exacerbated when working with genes other than the 16S rRNA or with sequences from hard-to-culture or yet uncultured organisms, as is usually the case of colonic microbes, making them inherently limited (116).

On the other hand, OTU-based methods do not rely on reference databases; they calculate a distance matrix among all query sequences and group them based on their similarity at a given threshold. Since grouping does not require previous taxonomic information, these OTU-based methods perform very well with poorly characterized microorganisms. OTU-based methods are not without faults, however. They are usually computationally exigent and prone to overestimation due to low-quality sequences, contamination, chimeras, etc. (117).

In turn, most OTU-clustering algorithms fall into two broad categories, hierarchical clustering (HC) and greedy heuristic clustering (GHC). HC and GHC differ in the methods for comparison of sequences and clustering into OTUs, their computational requirements, and the accuracy of the result. HC methods start by generating distance matrices that measure the distance between each pair of sequences in the dataset, either by multiple [e.g., Mothur (118)] or pairwise [e.g., ESPRIT (119)] sequence alignments, and then apply standard HC (single, complete or average linkage clustering) to group OTUs at a given threshold (usually, 97%). While debated (120), the use of multiple sequence alignment is preferred over pairwise alignments because it preserves positional homology across all sequences (121). The incorporation of the secondary structure of the 16S rRNA molecule into the alignment provides additional biological information that strengthens the confidence that positional homology is being conserved (122, 123). HC methods are computationally complex; however, several approaches have been devised to reduce their complexity and computer memory requirements (116, 119, 121), and software such as Mothur (from version 1.27.0) performs well with reasonable computer capacities.

Yet, computational requirements of HC algorithms can be a real headache in the analysis of many fecal samples; GHC algorithms have been developed to this end. They process input sequences one at a time, hence, avoiding the comparison of all pairs of sequences and the construction of a distance matrix (115). In GHC, the query sequence is compared against a set of seed sequences (or centroids) that are representative of existing clusters; if the similarity of the query and the seed sequences is above a given threshold (usually, 97%), the query sequence is assigned to the existing cluster, otherwise it becomes the seed of a new cluster or it is discarded. The seed sequences can be obtained either by generating them *de novo* [e.g., CD-HIT (124) or UCLUST (125)] or from a database of predefined centroids [e.g., UCLUST as implemented by QIIME (126, 127)]; the latter approach has the same limitations of other database-dependent methods, as discussed above. Furthermore, the centroid databases are constructed by clustering full-length sequences at a defined threshold; when used to cluster partial sequences, problems may arise. Some taxa may have identical sequences within a specific 16S sub-region, yet, they can be below the predefined threshold when the full-length sequence is considered; the opposite would also be true.

As with other steps in the workflow discussed here, there is a trade-off between complexity and accuracy. Different clustering methods can yield different results from identical datasets; their performance varies according to the complexity and the abundance ratio of the sequences in the dataset and the selected similarity threshold (117). Benchmark studies have consistently shown that methods such as complete linkage (HC), average linkage (HC), and CD-HIT (GHC) are robust to changing OTU thresholds and produce consistent clusters. On the other hand, single linkage (HC) produces OTUs that are not homogeneous and together with UCLUST (GHC) and UPARSE (GHC) have been shown to be very sensitive to threshold definitions and to have reproducibility issues, thus, in our opinion, their use should be less encouraged (115, 128, 129).

#### Taxonomic Assignment

In order to establish the biological significance of any intervention on the gut microbiome, it is usually desired to give a taxonomic classification to the previously detected OTUs. Several methods for the taxonomic assignment of 16S rRNA gene sequences are available and are based on different principles, such as k-mer count [SINA (130), RDP Bayesian classifier (88)], multiple sequence alignment [NAST (131)], BLAST [TUIT (132)], and machine learning algorithms [16S classifier (133)], among others. Although new algorithms continue to be developed, the RDP Bayesian classifier remains the most widely used tool for taxonomic assignment of 16S sequences; it provides taxonomic assignments from domain to genus, with confidence estimates for each assignment. The misclassification rate of short sequences varies approximately from 16 to 20% according to the dataset used to train the algorithm and the 16S rRNA gene region (114). As with others database-dependent methods, flaws in the databases will unavoidably lead to flaws in classification; fortunately, the approach used to label OTUs can reduce the error.

Regardless of the algorithm, OTUs can be classified either by assigning them the taxonomy of a representative sequence (127) or by classifying every sequence in the OTU and assigning the taxonomy by majority consensus (116). The former method can yield a less robust classification; if an OTU is composed of related sequences but with divergent taxonomies, the classification of a single sequence can lead to an erroneous classification of the entire OTU. Therefore, we recommended using majority-consensus taxonomy to the cost of a less detailed classification (genus, species).

### Copy Number Variation

A problem that arises when studying the gut microbiome is the difference in the number of copies of the 16S rRNA gene among species, which can range from a single copy up to 15 (134). This variation can lead to erroneous abundance assessment; at equal number of cells, taxa with few copies of the 16S rRNA gene have lower amplicon counts than taxa with more copies of the gene. Therefore, CNV can result in over or underestimation of microbial abundance. CNV has not deserved full attention; yet, it is of utmost importance since it can result in a biased description of the microbial community. Indeed, it has been suggested that bacterial diversity could be overestimated by a factor of 3 due to 16S CNV (135).

In microorganisms with known 16S rRNA gene copy number, CNV could be corrected by weighting read counts by the inverse of its gene copy number. However, the problem is more difficult to deal with in cases where the gene copy number is unknown. A possible solution in these cases is to use the value of a closely related organism (136). Another possibility is to place 16S reads on a phylogenetic tree and calculate gene copy number using phylogenetically independent contrasts (137, 138). While these methods have been shown to improve the measures of diversity and abundance of microbial communities, they rely on databases of 16S and sequenced genomes, which, as with phylotype-based clustering, lack information of uncultured and poorly cultured organisms. In cases of poorly studied deep evolutionary lineages (say, rare phyla), CNV correction is definitely an unsolved issue.

Although CNV can move away estimates of diversity from reality, it must be noted that researchers usually want to compare these estimates between treatments (e.g., obese vs. lean, vaginal delivery vs. C-section, probiotic vs. placebo). In other words, we look for relative changes in the abundance of OTUs A, B, and C; even if they would be badly estimated due to the assumption that they only have one 16S rRNA gene each, what is important is to see how populations change under different tested conditions. The take-home message from CNV is that we should emphasize more comparisons of the same OTU among samples than comparisons among OTUs within samples.

### Essential Information That Should Be Included when Describing a Microbiome Study

In order to guarantee reproducibility of results, we encourage researchers and journals to explicitly include and require the following technical information in microbiome publications: (I) DNA extraction method, including the type of extraction kit if one was used and modifications to the standard protocol proposed by the manufacturer; (II) description of how DNA contamination was controlled for (e.g., DNA extraction of negative controls); (III) 16S rRNA hypervariable region targeted including the nucleotide sequences of the primers used; (IV) sequencing technology employed; (V) description of sequencing error-rate assessment (e.g., was a mock community sequenced in parallel with the samples?); and (VI) description of *in silico* analyses (culling of dubious sequences, removal of chimeras, OTU clustering and taxonomy assignment, copy number variation correction), including the code or command lines with parameters used if appropriate.

## Conclusion

The study of the gut microbiome is revolutionizing medicine and science by allowing understanding how microbes are intimately involved in many physiological processes. The gut microbiome is shifting from an appealing object of study to a precision medicine target. NGS have enabled the possibility to gather the most impressive amount of microbiome data at costs and speeds that were unthinkable a decade ago. However, these technologies have introduced new challenges in data analysis that researchers must take care of. We have, here, discussed some of these challenges and suggested ways to control them using available tools (see Table 2 for our recommendations to reduce the impact of these pitfalls). Our hope is that, while a minimum information standard that unifies the procedures of microbiome studies is established, researchers implement rigorous analyses so that their results better represent the microbial communities under scrutiny. Only by making as stringent as possible analyses and by guaranteeing the transparency and reproducibility of microbiome analyses (139) we will give the field its first dose of “healthy skepticism” (140).

**Table 2:** **Recommendations to reduce the impact of biases introduced in the different steps of the analysis of microbial communities using the 16S rRNA gene**.

Step	Main challenge	Possible solution	Importance
DNA extraction	Uneven representation of the microbial community under scrutiny.	The use of a DNA extraction method that includes a bead-beating step results in a more comprehensive representation of the microbial community.	Moderate
	Differential representation of microbial communities due to differences in DNA extraction kits.	Direct comparisons should be carried only between studies using the same DNA extraction kit.	Moderate
	Contamination by microbial DNA from the DNA extraction and PCR reagents.	In order to reduce the risk of contamination, the starting biomass should be maximized. To control it, the samples must be processed in random order, the kit lots must be included as metadata and technical controls from the reagents must be sequenced.	Moderate
Multi-template PCR	Differences in the estimated phylogenetic diversity between hypervariable regions of the 16S rRNA gene.	The region that best approximates the phylogenetic diversity given by the whole gene should be selected. The V4 region has been shown to approximate the phylogenetic diversity given by the whole gene and to result in best taxonomy labeling.	Moderate
	Uneven coverage of different microbial taxa by the PCR primers.	Bioinformatic tools, such as SILVA TestPrime, allow the evaluation of primers, and the ones with the highest coverage rate for the taxa known to be present in the microbial community of interest should be selected.	Moderate
		The microbial coverage is maximized by using degenerate primers.	High
		Direct comparisons should be carried only between studies using the same set of primers.	Moderate
Amplicon sequencing by NGS	Sequencing platform selection.	The selection of the sequencing platform should be made prioritizing error rate over sequencing depth and read length.	High
	Assessment of the quality of the sequencing run.	The sequencing of a mock community allows the quality assessment of each individual amplicon sequencing run.	High
Culling of dubious sequences	Overestimation of diversity caused by spurious sequences.	Apply a stringent sequence denoising and curation procedures and assess their effectiveness by determining the final error rate using a sequenced mock community.	High
Chimera removal	Overestimation of diversity caused by non-existent organisms (chimeric sequences).	The use of database-free approaches, especially when studying poorly characterized environments, is encouraged.	Moderate
OTU clustering and taxonomy assignment	Overestimation of diversity caused by clustering algorithms.	Database-free OTU-based methods should be preferred over taxonomic-dependent (phylotyping) approaches.	Moderate
		If computationally possible, the use of hierarchical methods such as average or complete linkage should be used, otherwise, a heuristic method such as CD-HIT is suggested.	Moderate
	Erroneous taxonomic classification of OTUs.	The taxonomic assignment should be carried by majority consensus of the sequences within the OTU.	Moderate
Copy number variation	Over- or underestimation of diversity caused by erroneous abundance assessment.	While algorithms that correct CNV exist, they depend on whole genome sequence data, which may not be available for poorly described microorganisms, thus, their use is not encouraged	Low

## Author Contributions

JC-Z and JE devised, wrote, and made corrections to the manuscript.

## Conflict of Interest Statement

The authors declare that the research was conducted in the absence of any commercial or financial relationships that could be construed as a potential conflict of interest.
